# Emotional intelligence and mental health of high-performance competitive sports coaches: the chain mediating role of coping styles and resilience

**DOI:** 10.3389/fpubh.2026.1894961

**Published:** 2026-07-13

**Authors:** Fa Wang, Kerong Mou

**Affiliations:** 1 School of Leisure Sports, Chengdu Sport University, Chengdu, China; 2 College of Physical Education and Health, Aba Teachers College, Shuimo, China

**Keywords:** chain mediating effect, competitive sports coaches, coping styles, emotional intelligence, mental health, resilience

## Abstract

**Objective:**

To explore the relationship between emotional intelligence and mental health among high-performance competitive sports coaches, as well as the serial mediating effect of positive coping styles and resilience between the two variables, so as to provide a theoretical basis for improving coaches’ mental health.

**Methods:**

A questionnaire survey was conducted among 172 high-performance competitive sports coaches in Sichuan Province, China. Four measurement tools were adopted, including the Emotional Intelligence Scale, Symptom Checklist 90 (SCL-90), Simplified Coping Style Questionnaire and Resilience Scale. Data were analyzed via serial mediation analysis with Hayes’ PROCESS Macro Model 6.

**Results:**

Mental health of high-performance competitive sports coaches was significantly negatively correlated with emotional intelligence (*r* = −0.213) and resilience (*r* = −0.402) (*p* < 0.01), and significantly positively correlated with negative coping styles (*r* = 0.356, *p* < 0.01). Emotional intelligence showed a significant positive correlation with positive coping styles (*r* = 0.328) and resilience (*r* = 0.437) (*p* < 0.01). The mediation effect test indicated that three correlational indirect pathways linking emotional intelligence to mental health were identified in mediation models. Resilience exerted an independent mediating effect, and positive coping styles combined with resilience played a chain mediating effect. By contrast, the independent mediating effect of positive coping styles was not established, and the pathway from positive coping styles to mental health was statistically insignificant.

**Conclusion:**

Coaches’ emotional intelligence can predict their mental health, and higher emotional intelligence corresponds to better mental health status. Resilience acts as an independent mediator between emotional intelligence and mental health. Positive coping styles and resilience jointly exert a chain mediating effect. Specifically, emotional intelligence is correlated with greater use of positive coping styles and higher resilience, and all three variables jointly display correlational links to better mental health status.

## Introduction

As the core leaders of sports training, high-performance competitive sports coaches (hereafter referred to as coaches) undertake multiple responsibilities, including cultivating high-level athletes, striving for excellent competition results and inheriting sports spirits (1). Guided by the Olympic motto “Faster, Higher, Stronger – Together,” coaches are confronted with intensive training loads and tremendous performance pressure from competitions. Meanwhile, they need to handle various affairs such as athlete management, team coordination and career development, which keeps them under chronic high stress (2, 3). Although coaches commonly suffer from psychological problems such as anxiety, depression and emotional exhaustion (4, 5), their mental health issues have long been overlooked by researchers compared with athletes. Coaches’ mental health not only directly affects their coaching performance, but also exerts an influence on athletes’ psychological states (6). Therefore, an in-depth exploration of the influencing factors and underlying mechanisms of coaches’ mental health is of great practical significance for developing targeted mental health intervention programs, reducing the incidence of job burnout, and improving the quality of training and competition guidance for athletes.

Emotional intelligence (7), a core competency for individuals to perceive, understand and regulate their own and others’ emotions, serves as a critical psychological protective factor in high-pressure working environments. Existing studies have demonstrated that emotional intelligence is closely associated with coaches’ coaching effectiveness and leadership styles (8). Coaches with high emotional intelligence are more capable of managing negative emotions during training and competitions, optimizing coach-athlete interactions (9), and maintaining a lower level of job burnout (10). In a study on national cricket competitions, Crombie et al. (11) found that emotional intelligence scores could predict team athletic performance, which indirectly reflected the positive impact of emotional intelligence on coaches’ working status. After improving coaches’ emotional intelligence through online intervention training, Zajonz et al. (12) observed a remarkable increase in their coaching self-efficacy, further verifying the vital role of emotional intelligence in coaches’ career development. Nevertheless, most of the aforementioned studies (9–12) focus on the relationships between emotional intelligence and coaches’ coaching performance as well as leadership behaviors. Research concerning the direct association between emotional intelligence and mental health and its internal correlational pathways remains limited. Only a few studies have mentioned that emotional intelligence may correlate with mental health via stress coping correlates (11), yet relevant mechanism verification is still lacking.

Coping styles, divided into positive and negative coping styles (13), refer to individuals’ cognitive and behavioral strategies when confronting stress, and act as an important bridge linking emotional intelligence and mental health. In the field of competitive sports, positive coping styles such as problem-solving and social support seeking help coaches resolve conflicts and stress arising from training and competitions, while negative coping styles including avoidance and denial may aggravate their psychological burden (1). Hwang et al. (14) revealed that emotional intelligence shapes coaches’ leadership styles by influencing their selection of coping strategies, suggesting that coping styles may play a mediating role in the functional pathway of emotional intelligence. However, current research has not clarified whether coping styles exert an independent mediating effect between emotional intelligence and coaches’ mental health, and empirical evidence on this issue is insufficient.

Resilience is defined as individuals’ ability to adapt and recover when faced with adversity (15), and it is a key psychological resource for coaches to resist occupational stress and maintain sound mental health. Jowett et al. (16) pointed out that emotional intelligence and coach-athlete relationships jointly affect coaches’ job satisfaction, with resilience serving as a potential moderator in this process. Magrum et al. (17) confirmed that emotional intelligence has a significant positive effect on coaches’ coaching success, and this effect is likely realized by enhancing resilience. In addition, there is a close correlation between coping styles and resilience. The adoption of positive coping strategies can effectively improve individual resilience (18), and enhanced resilience in turn promotes the use of positive coping styles. The two variables may form a chain pathway affecting mental health. Previous studies have shown that positive coping styles can boost resilience by accumulating stress management experience (19), and emotional intelligence can positively predict positive coping styles (20). Accordingly, we hypothesize a chain pathway: emotional intelligence predicts coping styles, which in turn predicts resilience, and ultimately affects mental health. To date, few studies in sports science have systematically examined the chain mediating effects of coping styles and resilience between emotional intelligence and coaches’ mental health. This research gap hinders a comprehensive understanding of the mechanisms underlying coaches’ mental health. A thorough review of domestic and international literature identifies three limitations of prior research. First, most studies of coaches’ emotional intelligence center on coaching efficacy and leadership, with scarce investigations into the intrinsic mechanism connecting emotional intelligence to mental health. A handful of studies merely hypothesize an indirect role of coping styles yet lack empirical validation. Second, existing work only separately examines coping styles or resilience as isolated mediators and fails to build a positive coping-resilience chain mediation model, leaving the sequential pathway from emotional intelligence to mental health unelucidated. Third, nearly all empirical samples draw on Western coaches (21), and no localized research has tested this chain mediation mechanism among high-performance competitive sports coaches in Sichuan, China. Addressing these research gaps, the present study constructed a serial mediation model to test the independent and sequential mediating roles of positive coping and resilience, providing theoretical evidence for mental health interventions for domestic high-performance coaches.

This study built its conceptual framework based on two classic psychological theories. Per Fredrickson’s (22) broaden-and-build theory, coaches with high emotional intelligence regulate positive emotions, adopt positive coping strategies, accumulate psychological resources, strengthen resilience, and reduce mental distress. Under stress coping resource theory, emotional intelligence functions as a primary internal psychological resource that sequentially shapes coping choices and resilience to buffer occupational stress harming mental health. Supported by prior evidence that emotional intelligence predicts positive coping, positive coping boosts resilience, and resilience lowers psychological symptoms, four serial mediation hypotheses are proposed. This study proposes three research questions. First, can high-performance competitive sports coaches’ emotional intelligence significantly predict their mental health? Second, do positive coping styles and resilience separately mediate the association between emotional intelligence and mental health? Third, do positive coping styles and resilience together form a serial mediating pathway linking emotional intelligence to mental health? Given the above research status and deficiencies, this study constructs a chain mediation model and puts forward the following hypotheses: H1: Higher emotional intelligence is associated with better mental health among coaches. H2: Positive coping styles form an independent correlational mediating pathway linking emotional intelligence and coaches’ mental health. H3: Resilience mediates the relationship between emotional intelligence and mental health of high-performance competitive sports coaches. H4: Coping styles and resilience jointly form a serial correlational mediational pathway linking emotional intelligence and coaches’ mental health. This study aims to explore the correlation between emotional intelligence and mental health of high-performance competitive sports coaches and clarify the serial mediating mechanisms of positive coping styles and resilience between the two variables to provide empirical support for targeted mental health interventions for coaches.

## Materials and methods

### Participants

**Figure 1:**
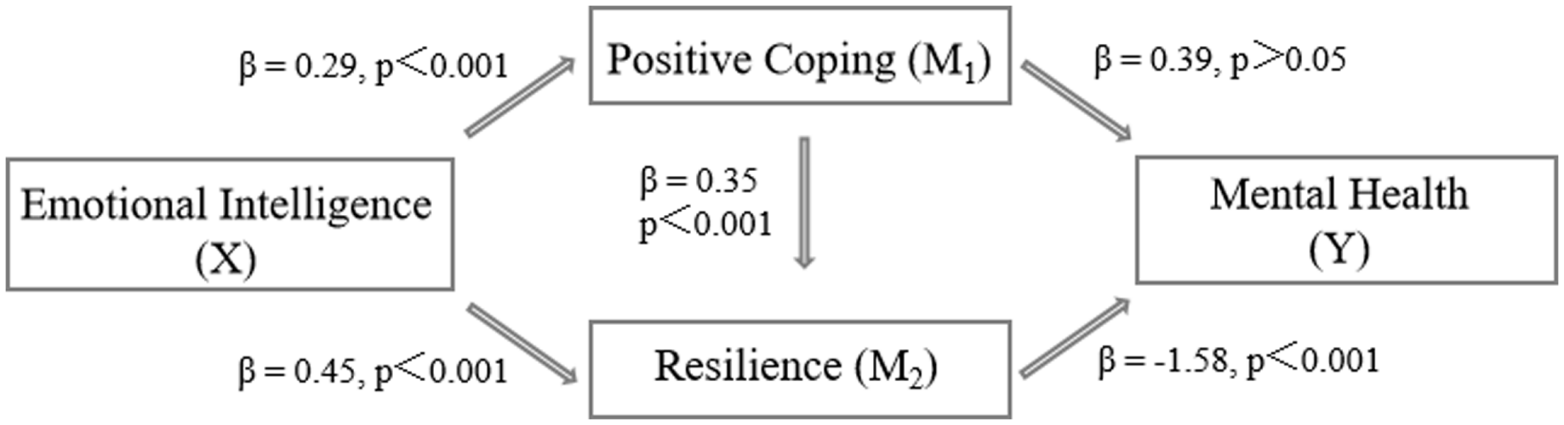
Chain mediating model of emotional intelligence, positive coping, resilience, and mental health.

This study was conducted in accordance with the Declaration of Helsinki and approved by the Ethics Committee for Human Experiments of Aba Teachers College (No: 202602). Participants were recruited from high-performance competitive sports coaches in Sichuan Province via random sampling. The sample size was determined based on the minimum requirements for mediation effect analysis. Referring to the sample size criteria for serial mediation models proposed by Hayes (23), the minimum required sample size for a four-variable chain mediation model is 160 participants. We further conducted *a priori* power analysis via G*Power 3.1. Setting medium effect size f^2^ = 0.15, *α* = 0.05, the calculated minimum sample size was 152. Our final valid sample of 172 exceeded this threshold, yielding statistical power above 0.80 to detect the hypothesized mediation effects. To account for questionnaire invalidity rate and ensure statistical power, a total of 180 coaches were initially recruited. All participants in this study were full-time coaches from professional sports teams at all levels in Sichuan Province, corresponding to Tier 3 (provincial/national training institutions) high-performance coaches under McKay’s (24) Participant Caliber Framework, with formal professional competitive coaching qualifications (Figure 1).

Inclusion criteria: having engaged in specialized competitive sports coaching for no less than 1 year; aged between 25 and 55 years; voluntary participation and signed informed consent. Exclusion criteria: diagnosed with mental disorders or receiving relevant treatment within the past six months; suffering from severe physical illnesses that may affect mental status; submitting incomplete questionnaires, logically contradictory answers or obviously perfunctory responses (e.g., selecting the same option for consecutive items).

#### Measurement tools

##### Emotional intelligence scale (EIS)

This scale was originally developed by Schutte et al. (25) and revised into the Chinese version by Shi (26). It consists of 33 items covering four dimensions: emotion perception, self-emotion management, others’ emotion management and emotion utilization. A 5-point Likert scale was adopted, ranging from 1 (totally disagree) to 5 (totally agree). A higher total score indicates a higher level of emotional intelligence. The scale has been widely validated among Chinese sports populations with sound construct validity, and the factor loading of each dimension exceeds 0.60 (26). In the present study, the Cronbach’s *α* coefficient of this scale was 0.886. This scale yielded composite reliability (CR) = 0.902 and average variance extracted (AVE) = 0.573 in the current sample, with AVE above 0.5 demonstrating adequate convergent validity.

##### Symptom checklist 90 (SCL-90)

Developed by Derogatis (27) and revised into the Chinese version by Zhou (28), this scale contains 90 items grouped into 10 factors including somatization, interpersonal sensitivity, anxiety and depression, which comprehensively reflect individuals’ mental health status. Responses were rated on a 5-point Likert scale from 1 (none) to 5 (severe). A higher total score suggests more prominent mental health problems. The revised Chinese version presents good construct validity and criterion-related validity among occupational groups, with correlation coefficients between each factor and the total score above 0.70 (28). The Cronbach’s *α* coefficient in this study was 0.839. The CR of SCL-90 was 0.871 and AVE = 0.526, meeting the standard for convergent validity.

##### Simplified coping style questionnaire (SCSQ)

This questionnaire was compiled in Chinese by Xie (29), comprising 20 items divided into two dimensions: positive coping (Items 1–12) and negative coping (Items 13–20). A 4-point Likert scale was used, with 0 (never adopt) to 3 (always adopt). Higher dimension scores indicate a greater tendency to adopt the corresponding coping styles. Its construct validity has been verified, and confirmatory factor analysis shows satisfactory model fit for the two dimensions (29). The Cronbach’s *α* coefficient of the questionnaire in this study was 0.879. The scale had CR = 0.895 and AVE = 0.551, showing acceptable convergent validity.

##### Connor-Davidson resilience scale (CD-RISC)

Developed by Connor and Davidson (30) and revised into the Chinese version by Yu (28), This 25-item scale covers three dimensions (tenacity, strength, optimism) and uses a 5-point Likert response scale from 1 (never) to 5 (almost always). A higher total score represents stronger resilience. The revised Chinese version has good construct validity among sports practitioners, with factor loadings of all three dimensions greater than 0.55 (31). The Cronbach’s *α* coefficient in this study was 0.868. The composite reliability was 0.884 with AVE = 0.542, confirming satisfactory convergent validity.

#### Questionnaire administration standard

The questionnaires were distributed offline in a targeted manner. To control common method bias, several strategies were implemented during questionnaire design and data collection: items were randomly arranged, and positively and negatively scored items were mixed. All participants answered anonymously and completed the questionnaires independently on site to reduce response bias. Prior to distribution, coaches were fully informed of the research purpose, questionnaire structure, estimated completion time and data confidentiality principles. It was clearly stated that the questionnaires were only used for academic analysis, all personal information would be fully anonymized, and there were no associated interests or potential risks. After participants fully understood the requirements and signed written informed consent forms, questionnaires were distributed on site.

Research staff stayed on site throughout the completion process to provide timely assistance and answer questions about item interpretation and response logic. Participants were reminded to fill in the questionnaires truthfully based on their actual situations and avoid biased or perfunctory answers. All questionnaires were collected immediately upon completion to ensure full process control. Collected questionnaires were screened for validity immediately afterwards. Invalid questionnaires were excluded according to the following criteria: missing key information, with three or more blank items in core scale dimensions or demographic sections; logical contradictions, such as identical answers for five or more consecutive items within a single scale, or inconsistent responses between positively and negatively scored items without reasonable explanation; obvious perfunctory responses with a completion time of less than 5 min.

Ultimately, 172 valid questionnaires were obtained, yielding an effective response rate of 95.56%. Harman’s single-factor test was conducted to further test common method bias. A total of 23 factors had eigenvalues greater than 1, and the first factor explained 31.72% of the total variance, which was below the critical threshold of 40%, indicating no severe common method bias in this study.

### Statistical analysis

All data were processed using SPSS 27.0. Descriptive statistics including means and standard deviations were first calculated for all variables. Pearson correlation analysis was used to examine the correlations among emotional intelligence, coping styles, resilience and mental health. Hayes’ PROCESS Macro Model 6 was used to test serial mediation effects to test the chain mediating effects. The Bootstrap method with 5,000 resamples was applied to calculate 95% confidence intervals (CIs). A mediating effect was considered significant if the 95% CI did not contain zero (1). Additional quality control analyses including common method bias test were also performed. The significance level was set at *α* = 0.05.

## Results

A total of 172 valid questionnaires were collected, with an effective response rate of 95.56%. The basic demographic characteristics of participants were as follows: 104 males (60.47%) and 68 females (39.53%). In terms of age, 76 coaches (44.19%) were aged 25–35 years, 64 (37.21%) were 36–45 years old, and 32 (18.60%) were 46–55 years old. Regarding coaching experience, 54 coaches (31.40%) had worked for 1–5 years, 70 (40.70%) for 6–10 years, and 48 (27.90%) for 11 years or longer. In terms of sports events, 82 coaches (47.67%) were engaged in ball games, 46 (26.74%) in track and field, 24 (13.95%) in combat sports, and 20 (11.63%) in other sports (see Table 1).

**Table 1:** Demographic characteristics of participants.

Demographic variable	Category	Number (*n* = 172)	Percentage (%)
Gender	Male	104	60.47
	Female	68	39.53
Age	25–35 years	76	44.19
	36–45 years	64	37.21
	46–55 years	32	18.60
Coaching experience	1–5 years	54	31.40
	6–10 years	70	40.70
	≥11 years	48	27.90
Sport type	Ball games	82	47.67
	Track and field	46	26.74
	Combat sports	24	13.95
	Other sports	20	11.63

Harman’s single-factor test confirmed no serious common method bias. Confirmatory factor analysis presented satisfactory model fit indices: χ^2^/df = 2.31, RMSEA = 0.06, CFI = 0.92, TLI = 0.91. The factor loadings of all scale items ranged from 0.58 to 0.89, demonstrating good convergent validity.

The average SCL-90 total score of participants was 148.35. This value was higher than the classic norm total score of 129.96 for ordinary Chinese adults (Jin et al., 1986), and consistent with the SCL-90 score range (142–155) reported among Sichuan elite competitive sports coaches (1). The clinical cutoff total score of SCL-90 is 160 (32), which indicates that coaches in this sample only exhibited mild subclinical psychological distress and did not reach the clinical threshold for mental disorders.

Table 2 presents the descriptive statistics and correlation analysis results of all variables. Emotional intelligence was significantly negatively correlated with mental health (total score of SCL-90) (*r* = −0.213, *p* < 0.01), indicating that coaches with higher emotional intelligence had better mental health status, which supported Hypothesis H1. Emotional intelligence was positively correlated with positive coping styles (*r* = 0.328, *p* < 0.01) and resilience (*r* = 0.437, *p* < 0.01), and negatively correlated with negative coping styles (*r* = −0.276, *p* < 0.01). Mental health showed significant negative correlations with positive coping styles (*r* = −0.245, *p* < 0.01) and resilience (*r* = −0.402, *p* < 0.01), and a significant positive correlation with negative coping styles (*r* = 0.356, *p* < 0.01). Positive coping styles were positively associated with resilience (*r* = 0.341, *p* < 0.01), while negative coping styles were negatively associated with resilience (*r* = −0.207, *p* < 0.05).

**Table 2:** Descriptive statistics and correlations among variables (*n* = 172).

Variable	M	SD	1	2	3	4
1. Emotional intelligence	128.63	11.47				
2. SCL-90 total score	148.35	61.28	−0.213**			
3. Positive coping	28.74	5.32	0.328**	−0.245**		
4. Negative coping	15.62	4.18	−0.276**	0.356**	−0.189*	
5. Resilience	68.79	14.83	0.437**	−0.402**	0.341**	−0.207*

*p < 0.05, **p < 0.01.

The chain mediating effects were tested via Model 6 of the PROCESS macro, and the results are shown in Table 3. Taking emotional intelligence as the independent variable, positive coping styles and resilience as mediating variables, and mental health as the dependent variable, the regression analysis revealed that emotional intelligence was positively correlated with positive coping styles (*β* = 0.29, *p* < 0.001) and resilience (*β* = 0.45, *p* < 0.001). Positive coping styles showed significant positive correlations with resilience (β = 0.35, *p* < 0.001). Resilience displayed significant negative correlations with mental health (β = −1.58, *p* < 0.001). After controlling for mediating variables, the direct correlational pathway between emotional intelligence and mental health was non-significant (β = −0.19, *p* = 0.571), suggesting that correlations between emotional intelligence and mental health operate mainly through indirect correlational pathways.

**Table 3:** Regression analysis of study variables (standardized β coefficients, *n* = 172).

Outcome variable	Predictor	R	R^2^	F	β	t	*p*
SCL-90 total score	Emotional intelligence	0.21	0.04	7.53	−0.213	−2.74	0.008
Positive coping	Emotional intelligence	0.33	0.11	10.67	0.29	3.27	0.002
Resilience	Emotional intelligence	0.44	0.19	19.82	0.45	4.45	<0.001
	Positive coping	0.49	0.24	13.15	0.35	3.63	0.001
SCL-90 total score	Emotional intelligence	0.41	0.17	14.86	−0.19	−0.57	0.571
	Positive coping				0.39	1.05	0.298
	Resilience				−1.58	−6.37	0.000

The decomposition of mediating effects is displayed in Table 4. The total indirect effect was −0.78 (95% CI [−1.18, −0.45]). The independent mediating effect of resilience (−0.71) served as the primary pathway, and the chain mediating effect of positive coping styles and resilience (−0.16) served as the secondary pathway. Indirect Effect 1 (emotional intelligence to positive coping styles to mental health) had an effect value of 0.09 (95% CI [−0.07, 0.34]), with zero included in the confidence interval, indicating that the independent mediating effect of positive coping styles was not established. Thus, Hypothesis H2 was rejected. Indirect Effect 2 (emotional intelligence through resilience to mental health) was −0.71 (95% CI [−1.08, −0.42]), excluding zero, which verified the independent mediating role of resilience and supported Hypothesis H3. Indirect Effect 3 (emotional intelligence through positive coping styles to resilience to mental health) was −0.16 (95% CI [−0.28, −0.06]), excluding zero. This result confirmed the chain mediating effect of positive coping styles and resilience, so Hypothesis H4 was supported.

**Table 4:** Decomposition of total, direct, and indirect effects (unstandardized raw effect values; unit: SCL-90 total score per one unit of emotional intelligence scale score, *n* = 172).

Effect type	Effect value	Bootstrap SE	BootCI lower	BootCI upper
Total effect	−0.21	0.078	−0.366	−0.060
Direct effect	−0.19	0.333	−0.848	0.468
Total indirect effect	−0.78	0.205	−1.180	−0.450
Indirect effect 1	0.09	0.103	−0.070	0.340
Indirect effect 2	−0.71	0.176	−1.080	−0.420
Indirect effect 3	−0.16	0.058	−0.280	−0.060

Indirect effect 1: Emotional Intelligence to Positive Coping to SCL-90 total score; Indirect effect 2: Emotional Intelligence to Resilience to SCL-90 total score; Indirect effect 3: Emotional Intelligence to Positive Coping to Resilience to SCL-90 total score. Direct, indirect and total indirect effects in this table are unstandardized raw regression coefficients; total effect r = −0.213 is standardized zero-order correlation coefficient.

## Discussion

### Relationship between emotional intelligence and mental health of competitive sports coaches

This study verified Hypothesis H1. Emotional intelligence was significantly negatively correlated with coaches’ mental health, and it exerted no significant direct effect on mental health. Instead, its influence was realized via two indirect pathways: the independent mediating effect of resilience, and the chain mediating effect of positive coping styles and resilience. In other words, coaches with higher emotional intelligence tended to have better mental health, which was consistent with findings of previous studies (9–11).

As a vital psychological resource, coaches with higher emotional intelligence tend to better recognize and regulate their own and athletes’ emotions under high-pressure training and competition circumstances, alongside lower self-reported emotional exhaustion (10). When facing stressful events such as competition failures and conflicts in athlete management, coaches with high emotional intelligence tend to maintain emotional stability, solve problems rationally and report lower levels of anxiety and depression (11). This conclusion was also supported by Ugrenovic et al. (33), who found that high-level competitive sports coaches with higher emotional intelligence reported lower job burnout and stronger psychological adjustment ability. In addition, coaches with superior emotional intelligence generally possess better interpersonal communication skills. They can build sound relationships with athletes and administrative departments to obtain more social support, which correlates with more favorable self-reported mental health outcomes.

### Mediating effect of coping styles

Different from the significant independent mediating effect of resilience, the independent mediating role of positive coping styles was not validated in this study, and Hypothesis H2 was not supported. This finding was inconsistent with the results among college students reported by Qiang et al. (34). Several reasons may account for this discrepancy. Firstly, coaches face unique stressors such as intensive performance pressure and long athlete cultivation cycles. Correlations linked to positive coping styles are bounded by various objective factors, such that a single coping variable cannot fully account for the correlational link between emotional intelligence and mental health (35). Secondly, under the cultural background of competitive sports, occupational stress indicators show weak standalone correlations with positive coping styles. Multiple psychological resources such as resilience and social support share overlapping correlational connections with stress and mental health. It indicates that correlational links between emotional intelligence and mental health operate through a multi-variable serial pathway rather than a simple single-variable correlational mediator. Thirdly, mental health is a complex multi-dimensional construct with correlational connections to social support, professional identity and other variables. A single mediating variable cannot fully explain the association between emotional intelligence and mental health (36).

### Mediating effect of resilience

Hypothesis H3 was supported in this study, confirming the independent mediating effect of resilience between emotional intelligence and coaches’ mental health. This result is in line with the research of Xing et al. (37) on the pathway from empathy to resilience via emotional intelligence, and also verifies the viewpoint proposed by Magrum et al. (17) that emotional intelligence shares positive correlations with coaches’ career success alongside positive correlations with resilience.

Emotional intelligence was significantly positively correlated with resilience and shares strong correlational links with this psychological resource. Coaches with higher emotional intelligence tend to better regulate emotions and integrate resources when confronting adversity, alongside higher self-reported psychological adaptability. Coaches with high emotional intelligence can perceive changes in their psychological states more sensitively. When encountering setbacks such as training bottlenecks and competition losses, they tend to maintain psychological balance through positive self-suggestion and emotional regulation, alongside higher self-reported resilience (12). Coaches with strong resilience report greater ability to withstand setbacks, alongside more positive working and mental states in difficult contexts and more favorable mental health indicators (38). This correlational pattern indicates that resilience shares strong links with coaches’ mental health outcomes. Relevant training programs targeting emotional intelligence and emotion regulation may correlate with higher self-reported resilience levels.

### Chain mediating effect of positive coping styles and resilience

Hypothesis H4 was verified. Positive coping styles and resilience played a significant chain mediating role between emotional intelligence and coaches’ mental health. Specifically, emotional intelligence correlates with greater adoption of positive coping styles, which in turn correlates with higher resilience; all three variables together display favorable correlational links with mental health indicators. This finding aligns with correlational patterns observed among enterprise employees by Gong (39), and further demonstrates that this serial correlational pathway of positive coping styles and resilience also exists within high-stress coach populations.

To be specific, coaches with high emotional intelligence can better understand the nature of stressful events and are more inclined to adopt positive coping strategies such as problem-solving and seeking social support (34). Consistent use of positive coping styles correlates with richer stress management experience, higher self-confidence, and elevated resilience scores (1). According to Fredrickson’s broaden-and-build theory of positive emotions (22), short-term psychological improvements brought by positive coping styles can be accumulated into long-term psychological resources and further strengthen resilience, enabling coaches to recover psychologically more quickly when facing similar stress afterwards. The identified serial correlational pathway reveals complex correlational mechanisms linking emotional intelligence and coaches’ mental health, and provides an important reference for developing targeted mental health intervention programs.

### Limitations and future prospects

This study has several limitations. First, all participants were recruited exclusively from Sichuan Province, which introduces sampling bias and limits the external validity of our findings. Second, the cross-sectional design only identifies variable correlations and cannot confirm causal directions between emotional intelligence and resilience. Third, all data were collected via self-report scales, which may bring subjective response bias. Fourth, we omitted demographic and occupational covariates (gender, age, coaching experience, sport type) in regression models, leaving potential residual confounding unaccounted for. Fifth, potential moderators such as sport category and coaching rank were not included to test their regulating effects. Besides, this paper only analyzed positive coping styles in the mediation model; although negative coping correlated significantly with core variables, its underlying mechanism remains unclarified.

For future research, multi-regional samples of coaches across various sports should be recruited to enhance external validity. Longitudinal tracking spanning 1–2 years is suggested to capture psychological fluctuations throughout training and competition cycles and validate causal pathways. Researchers may add more moderators (social support, professional identity, training load) to build a more comprehensive predictive model. Intervention trials can also be carried out to develop practical mental health promotion strategies for coaches.

## Conclusion

Coaches’ emotional intelligence displays significant correlations with their mental health, and higher emotional intelligence corresponds to better self-reported mental health status. Resilience forms an independent correlational mediational pathway linking emotional intelligence and mental health. Positive coping styles and resilience jointly form a serial correlational mediational pathway. Specifically, emotional intelligence correlates with greater use of positive coping styles, which in turn correlates with higher resilience; all three variables collectively show favorable correlational links to better mental health status.

## Data Availability

The raw data supporting the conclusions of this article will be made available by the authors, without undue reservation.
